# Prehospital neurological emergencies– a survey on the state of prehospital neurological assessment by emergency medical professionals

**DOI:** 10.1186/s12873-024-01076-w

**Published:** 2024-09-11

**Authors:** Vesta Brauckmann, Dominica Hudasch, Pascal Gräff, Torben Riecke, Gökmen Aktas, Jorge Mayor, Christian Macke

**Affiliations:** 1 Department of Trauma Surgery, Hannover Medical School, Carl-Neuberg-Straße 1, D-30625 Hannover, Germany; 2 Department of Neurology, Hannover Medical School, Carl-Neuberg-Straße 1, 30625 Hannover, Germany

**Keywords:** Prehospital emergency medicine, Neurological examination, EMS, Stroke, Emergency medicine

## Abstract

**Background:**

Neurological emergencies are one of the major diagnosis groups in the Emergency Medical Services (EMS) with the highest rate of misdiagnosis. Despite the knowledge of time sensitivity and the importance of prehospital factors, prehospital delay is common. Although several stroke triage scales have been developed, a gold standard in the prehospital setting is lacking.

**Objectives:**

Our aim was to evaluate the perception of neurological emergencies by EMS personnel and to identify current problems, difficulties and opportunities for improvement in the prehospital management of stroke, seizure, non-specific neurological symptoms, and paediatric neurological emergencies.

**Methods:**

The study was conducted as an online survey through SoSci Survey and was made available from March 1st to June 30th 2023 to all personnel working in emergency medical services. The access link was distributed through snowballing, social media, and through a QR code on a promotional poster. The survey was completed anonymously. The final survey consisted of 30 questions in German on the topics of neurological emergencies, general neurological assessment, specific neurological examination including paediatric assessment, stroke, and seizures, and finally suggestions for improvement.

**Results:**

The largest group of participants were paramedics, who estimated to encounter neurological emergencies at a general rate of 20–60%. When unease was felt, the main reasons were ambiguity of symptoms and insufficient admission capacity of hospitals. The biggest challenges were highly varied. Almost 80% of participants assumed that the neurological assessment would be omitted in difficult patient groups such as demented, intoxicated or children. 75% felt uncomfortable making a paediatric assessment, 50% were unfamiliar with the Paediatric Glasgow Coma Scale.

**Conclusions:**

Support through more standardized practical training and defined, uniform guidelines is needed. There was also a clear need for peer collaboration, feedback and case sharing. Digitalization, the usage of telemedicine and updated versions of the documentation protocols including paediatric adaptations to current guidelines could further improve current neurological assessment in the prehospital setting.

**Supplementary Information:**

The online version contains supplementary material available at 10.1186/s12873-024-01076-w.

## Introduction

The main symptom and diagnosis groups in Emergency Medical Services (EMS) include neurological, cardiovascular, pulmonary, and trauma, with a recent trend showing an increase in non-specific diagnoses [1–3]. However, the lowest agreement between prehospital and discharge diagnoses by emergency physicians, at 80%,has been observed for neurological disorders, and the same rate of agreement was found for paramedics in neurological emergencies [4, 5]. Diagnosis in neurological emergencies is known to be time-sensitive, particularly in the triage of stroke [6–11].

Despite the critical time frame, a prehospital delay of up to 73% was observed [9]. This delay results in a significant majority of patients not receiving the indicated thrombolytic therapy, leading to an increased risk of death [7, 12]. The reasons described in literature are multifactorial, and include contextual and behavioural factors, with a focus on the deficiencies in the recognition of neurological emergencies by medical professionals [7, 9, 12, 13].

The problem of stroke detection is a major topic in the field of prehospital neurological assessment research, with a multitude of different scales being developed [6]. For stroke-specific screening there stroke-detection scales, such as FAST (BE-FAST, FAST-ED, G-FAST) or the Cincinnati Prehospital Stroke Scale (CPSS), and there are stroke-severity scales, such as the 3-item stroke scale (3I-SS), the Austrian Prehospital Stroke Scale (APSS) and the Rapid Arterial Occlusion Evaluation scale (RACE).The shortened National Institute of Health Stroke Scale for EMS (sNIHSS-EMS) has been designed to include detection and severity [6]. Additionally, there are scales for assessing consciousness, such as the Full Outline of UnResponsiveness (FOUR) or the Glasgow Coma Scale (GCS) [6]. However, a neglected and emerging area is the practicability and the teaching of these scores in a prehospital setting due to the absence of a gold standard [14, 15].

Few reasons have been identified for the misdiagnosis of neurological emergencies, but the difficulty of obtaining a medical history for example from elderly or neurologically impaired patients has been described as a major factor [4]. Possible solutions for the initial diagnosis have focused on public education and the management by hospital emergency physicians rather than EMS-providers [16]. There are even fewer publications addressing the prehospital management of non-stroke neurological emergencies, including common but unspecific symptoms such as headache, dizziness, weakness, and seizures in adults or children [16]. It is known that the prehospital management can influence patient outcome and mortality, which is true for seizures as it is for cerebrovascular emergencies [17, 18]. In general, the need for specific training and the importance of a two-way communication with neurological specialists have been identified for paramedics [18, 19].

Our aim was to assess the perception of neurological emergencies by EMS personnel and to identify current problems, difficulties and opportunities for improvement in the prehospital management of stroke, seizure, non-specific neurological symptoms, and paediatric neurological emergencies. We considered it necessary to not only include the commonly discussed stroke triage but also the practice of neurological assessment prior to the diagnosis, management, and evaluation of seizures, modifications for paediatric patients, and documentation.

## Methods

### Study design

This study was conducted as an online survey on neurological assessment in a prehospital setting. The survey was available for four months, from March 1st to June 30th, 2023, and was open to all personnel working in EMS. The survey was conducted in German using the SoSci Survey online tool Version 3.4.17 (SoSci Survey GmbH, Munich, Germany). The survey link (https://webext.mh-hannover.de/soscisurvey/neuro-rd/) was distributed through snowball sampling, social media, and a QR code on promotional posters. These posters were displayed at the entrance to the emergency department and in the emergency rooms of a German tertiary care hospital (Supplementary Data 2).Participants were informed about the study’s purpose, data storage, and security before starting the questionnaire. Consent was obtained digitally, with participation being voluntary and anonymous,, and no compensation was provided. It was possible to skip questions, except for indicating the type of work in the EMS.

### Questionnaire development

The questionnaire was developed by an interdisciplinary team of emergency physicians and neurologists from a level I trauma centre. The English translation of the German version is provided as Supplementary Data 1. Items were individually identified based on the current documentation protocol, [20]. algorithms and assessment tools for paramedics and emergency physicians, [6, 21, 22]. current literature, [23–27]. and personal experience in the prehospital environment.

The final questionnaire comprised 30 items, categorized into basic demographics, neurological emergencies, general neurological assessment, specific neurological examination including paediatric assessment, prehospital stroke assessment, seizure management, and practicality of current documentation with suggestions for improvement.

Demographic items were nominal. Depending on the type of question, items were designed as six-point Likert-Scales ranging (e.g. from “very secure” to “very insecure”), as nominal scales for frequency distribution or as dichotomous scales (Yes/No with the alternative “I don’t know / not specified”). Finally, some items were based on a free-text responses.

### Statistical analysis

The statistical analysis of frequencies and distribution was performed using SPSS Statistics 28.0 (IBM, Armonk, New York, USA). All responses were included in the analysis.

## Results

During the four-month period there were 760 visits to the survey. 319 participants completed the first mandatory question, and 201 participants completed all pages. Of the 319 participants, paramedics were the largest group 41%. Emergency Medical Technicians (EMT) were represented with 38%, and emergency physicians with 6%. Other participants included paramedics in training, paramedics who graduated before 2013, and nurses specialising in emergency medicine. About a third (32.6%) of the participants had been working in EMS for less than three years, 25% between 3 and 5 years, and 38% more than 5 years. The main type of emergency vehicle was an ambulance with 78%. The second largest group was the emergency physician’s vehicle with 14%. The rescue helicopter and mobile intensive care unit were represented with 4%. See Table 1. Basic Demographic Data.

**Table 1:** Basic Demographic Data of 319 participants. EMS = Emergency Medical services

**Profession**	***N*** ** = 319 (100%)**
Emergency Physician	18 (5.6%)
Paramedic	130 (40.8%)
Emergency Medical Technician	122 (38.2%)
Other	41 (12.9%)
Not answered	8 (2.5%)
**Years of Experience in EMS**	
< 3 years	104 (32.6%)
3–5 years	81 (25.4%)
5–10 years	54 (16.9%)
> 10 years	66 (20.7%)
Not answered	14 (4.4%)
**Type of Emergency Vehicle**	
Rescue Helicopter	14 (4.4%)
Emergency Physicians Vehicle	45 (14.1%)
Ambulance	248 (77.7%)
Mobile Intensive Care Unit	14 (4.4%)
Other	53 (16.6%)

In general, over 90% of participants estimated that they encounter neurological emergencies about 20–40% of all callouts. 8% even estimated a higher frequency of over 60% of the time. Less than 10% and none of the emergency physicians felt at unease when confronted with a neurological emergency. Reasons were given in free text, with the majority feeling uncomfortable because of ambiguous or unclear systems and complex and varied neurological diagnoses. The other main reason was lack of capacity and refusal of hospitals to admit patients, which did not guarantee rapid care. Other reasons were lack of expertise and uncomprehensive medical history. *(*Table 2. *Subjective assessment of the prehospital neurological examination process)*

The biggest challenge in neurological emergencies was answered in free text form. 25% named the differentiation between ischaemic stroke and intracranial haemorrhage as the biggest challenge. For less than 20%, the biggest challenge was respectively unclear unconsciousness, and status epilepticus. For another 10% or less, the answer was unclear vertigo or traumatic brain injury. Other responses (< 5%) were headache, transient ischaemic attack, focal deficits, acute episodes of chronic diseases, intoxication, psychiatric emergencies, meningitis or encephalitis, dementia, paediatric emergencies, and unknown symptoms.

Almost 90% of participants felt confident in carrying out a neurological assessment. More than 90% reported having a neurological assessment scheme they can use. However, only three quarters were familiar with standard operating procedures for the neurological assessment. See Table 2. Subjective assessment of prehospital neurological examination process.

**Table 2:** Subjective assessment of prehospital neurological examination process

**How often do you encounter neurological emergencies?**	*N* = 227
20%	135 (59.5%)
40%	73 (39.2%)
60%	16 (7%)
> 80%	3 (1.3%)
**Do you feel a sense of unease when dispatched for a neurological emergency?**	*N* = 224
Yes* (Free Text Option)	19 (8.5%)
No	205 (91.5%)
**Rate the quality of your general neurological examination? From 1 (very good) to 6 (insufficient)**	*N* = 225
1	10 (4.4%)
2	102 (45.3%)
3	88 (27.6%)
4	24 (7.5%)
5	1 (0.4%)
6	0
**How secure do you feel while performing a neurological assessment?**	*N* = 225
Very secure	15 (6.7%)
Secure	84 (37.3%)
Rather secure	99 (44%)
Rather insecure	26 (11.6%)
Insecure	1 (0.4%)
**Do you have a fixed scheme you can use for performing a neurological examination?**	*N* = 224
Yes	203 (90.6%)
No	13 (5.8%)
I don’t know	8 (3.6%)
**Are you familiar with any standard operating procedures (SOPs) for prehospital neurological assessment?**	*N* = 225
Yes	171 (76%)
No	30 (9.4%)
I don’t know	24 (10.7%)

In terms of general neurological assessment, over 80% of participants considered the FAST score (face, arms, Speech, Time) or facial expressions and speech individually, as well as GCS, orientation, medical history and pupillary light response to be the most important items. Other items (see table 3) were less frequently considered relevant to the general neurological examination in a prehospital setting. This is consistent with the frequency of assessment of patient’s orientation. Almost 95% of the participants reported to assess orientation regularly. As for the pupillary light response more than 95% of the participants paid most attention to the size, isocoria and direct light reflex. Shape of and consensual light reflex were of secondary importance. See table 3. General neurological assessment.

**Table 3:** General neurological assessment. GCS = Glasgow Coma Scale

**What is relevant for you in your GENERAL neurological assessment?**	*N* = 206 (100%)
GCS	94.2%
Patient History	92.7%
Pupillary Light Response	92.2%
Speech	91.7%
Orientation	88.8%
FAST (Facial drooping, Arm weakness, Speech difficulties, Time to call emergency services)	87.4%
Facial Expressions	80.1%
Arm Drift Exam	78.2%
Asymmetry	73.8%
Sensory Function	60.7%
Circulatory, Motor, Sensory Testing	57.3%
AVPU (Alert, Verbal Response, Pain, Unresponsive)	57.3%
Strength Grade	51.5%
Visual Activity	26.2%
Reflexes	22.8%
Pathological Reflexes	19.9%
FOUR (Full Outline of UnResponsiveness)	7.3%
Other	5.8%
**What do you pay attention to when assessing pupils?**	***N*** ** = 206 (100%)**
Size	98.1%
Direct light reflex	96.6%
Isocoria	95.1%
Shape	84.0%
Consensual light reflex	57.8%
Other	5.8%
**How regularly do you evaluate the patient’s orientation(person**,** time**,** place and situation)?**	*N* = 208
Very regularly	92 (44.2%)
Regularly	80 (38.5%)
Rather regularly	25 (12%)
Rather unregularly	10 (4.8%)
Unregularly	1 (0.5%)

And although general neurological assessment appears to be routine, participants estimated that in > 75% the assessment is forgone in difficult patient groups, such as demented patients, intoxicated patients, or children. Less than 50% of participants confirmed that they knew how to perform a neurological examination in patients with altered consciousness, in the subgroup of emergency physicians the number was > 90%. The majority with > 70% felt insecure about performing a paediatric neurological assessment. In addition, only half of participants were familiar with the paediatric Glasgow Coma Scale and would change their approach for assessing children.

Responses to the questions about seizures were mostly uniform. > 90% knew the difference between focal and generalised seizures. Almost 90% knew how to distinguish between a seizure and a status epilepticus. Not uniformly answered was the question whether every seizure should be treated. See Table 4. Neurological Assessment in specific groups.

**Table 4:** Neurological Assessment in specific groups

**How often do you get the impression that with “difficult” patients no neurological assessment takes place? (E.g. Demented or intoxicated patients, children)**	*N* = 207
Very often	15 (7.2%)
Often	58 (28%)
Rather often	89 (43%)
Rather rarely	33 (15.9%)
Rarely	11 (5.3%)
Very rarely	1 (0.5%)
**Do you know how to perform a neurological examination on patients with impaired consciousness?**	*N* = 207
Yes	84 (40.6%)
No	96 (46.4%)
Not specified	27 (13%)
**How secure do you feel in performing a paediatric neurological examination?**	*N* = 206
Very secure	2 (1%)
Secure	16 (7.8%)
Rather secure	34 (16.5%)
Rather insecure	93 (45.1%)
Insecure	48 (23.3%)
Very Insecure	13 (6.3%)
**Do you change your neurological assessment when examining children?**	*N* = 207
Yes	117 (56.5%)
No	49 (23.7%)
Not specified	41 (19.8%)
**Are you familiar with the Paediatric Glasgow Coma Scale?**	*N* = 208
Yes	105 (50.5%)
No	90 (43.3%)
Not specified	13 (6.3%)
**Have you ever seen a status epilepticus?**	*N* = 201
Yes	163 (81.1%)
No	31 (15.4%)
I don’t know	7 (3.5%)
**Do you know what the difference is between a seizure and a status epilepticus?**	*N* = 201
Yes	178 (88.6%)
No	14 (7.0%)
I don’t know	9 (4.5%)
**Do you know the difference between focal and generalized seizures?**	*N* = 201
Yes	187 (93%)
No	9 (4.5%)
I don’t know	5 (2.5%)
**Do you need to treat every epileptic seizure?**	*N* = 200
Yes	62 (31%)
No	119 (59.5%)
I don’t know	19 (9.5%)

The questionnaire also asked about four specific stroke assessment scales. The FAST test was used by more than half of the participants and was known by more than 80%. The Cincinnati Prehospital Scale, the Rapid arterial occlusion evaluation and other tests such as the National Institute of Health Stroke Scale were known by less than 15% and used by less than 10%. In the subgroup analysis for stroke severity scales 23% of emergency physicians vs. 8% of paramedics and EMTs used the RACE scale. Stroke detection with the FAST test was used equally often by physicians and paramedics. Interestingly, the isolated parameters of the FAST test such as speech, limb motor function and facial symmetry were considered relevant in over 90% of participants. Less relevant, with < 85% were coordination, responsiveness and consciousness. Sensory function and oculomotor function were considered less important in stroke assessment and general neurological examination. Other items added were glucose level, breathing pattern, balance, and visual acuity (BE-FAST or Gaze-FAST), nystagmus and ocular deviation. See table 5. Specific Stroke Assessment.

**Table 5:** Specific stroke assessment

**What is relevant for you in a specific stroke assessment?**	*N* = 203 (100%)
Speech	98.5%
Motoric Function of the Extremities	96.6%
Facial Symmetry	95.1%
Coordination	88.2%
Obeying of Commands	86.7%
Orientation	86.2%
Consciousness	85.7%
Sensory Function	71.4%
Occulomotoric	61.6%
Other* (Free Text Option)	5.4%
**Are you familiar with specific tests / scales for stroke assessment? If yes**,** do you use them?**	***N*** ** = 203 (100%)**
FAST-Test (Facial Drooping, Arm weakness, Speech difficulties, Time to call emergency Services)	
*Known*	84.2%
*Used*	60.6%
RACE-Scale (Rapid Arterial Occlusion Evaluation)	
*Known*	7.9%
*Used*	5.9%
CPSS-Scale (Cincinnati Prehospital Stroke Scale)	
*Known*	11.8%
*Used*	6.4%
Other specific tests (e.g. NIHSS-Scale National Institute of Health Stroke Scale)	
*Known*	11.8%
*Used*	3.4%

Finally, we asked the remaining active participants (*N* = 197) if they considered a neurological assessment in a prehospital setting useful, and almost all participants agreed. 30% experienced poor correlation between assessment and documentation. The documentation form was considered adequate by almost 80%. The free text suggestions for improvement included the paediatric Glasgow Coma Scale, update / addition for stroke triage (e.g. NIHSS, BE-FAST) and other scores or even acronyms such as PERRLA (pupils, equal, round, reactive to light, accommodation), inclusion of (peripheral) sensitivity, inclusion of aphasia, amnesia and orientation, and a defined field for callback numbers of family members. The majority suggested a larger space for free-form documentation or the change to electronic documentation. Over 75% of participants clearly expressed a desire for more training. See table 6. Current documentation and future perspectives.

**Table 6:** Current documentation and future prospects

**How useful do you find a prehospital neurological assessment?**	*N* = 197
Very useful	101 (51.3%)
Useful	75 (38.1%)
Rather useful	18 (9.1%)
Rather useless	3 (1.5%)
**In your experience; How high is the correlation between the documentation and the actually performed examination?**	*N* = 197
Very high	16 (8.1%)
High	51 (25.9%)
Rather High	72 (36.5%)
Rather Low	42 (21.3%)
Low	15 (7.6%)
Very low	1 (0.5%)
How suitable do you think is the documentation for neurological emergencies? *If unsuitable: Free Text Option	*N* = 186
Very suitable	5 (2.7%)
Suitable	89 (47.8%)
Rather suitable*	53 (28.5%)
Rather unsuitable*	31 (16.7%)
Unsuitable*	8 (4.3%)
Very unsuitable*	8 (4.3%)
**Would you want more training initiatives for a prehospital neurological assessment?**	*N* = 199
Yes	153 (76.9%)
No	4 (2%)
Maybe	39 (19.6%)
I don’t know	3 (1.5%)

For the last question of the questionnaire, we left room for non-default answers. When asked what type of support was considered useful for further improvement, 30% responded. Of these, the majority focused on standardized practical training involving all EMS-personnel. Almost 20% felt that consistent, defined, and uniform guidelines and algorithms were necessary for further improvement. Around 10% considered peer feedback and collaboration to be most useful. 5% or less responded with the need to improve and update protocols including the lack of paediatric assessment, telephone support, implementation of digitalization and regular case discussions.

## Discussion

In our survey, participants encountered neurological emergencies in 20 to 40% of dispatches. Only a minority of participants felt uncomfortable with neurological cases, which was attributed not only to the wide range of neurological conditions or incomplete medical histories, but also to the lack of hospital admission capacity and resulting discussions and refusal of care. The free-text replies revealed a high frustration levels due to hospitals frequently refusing care. The impact of hospital capacities on the care of patients with acute neurological conditions has not yet been sufficiently reflected in literature [28].

Most participants were confident in performing a neurological assessment using a personal scheme. They claimed to be able to differentiate between a focal and generalized seizure and to distinguish between a seizure and a status epilepticus. However, nearly 80% admitted to shortening their assessment or not performing it at all in difficult patient groups, such as demented elderly patients, intoxicated patients, or paediatric patients. Moreover, 60% of participants did not know how to examine patients with impaired consciousness, and only half were familiar with the paediatric adaptation of the Glasgow Coma Scale. This aligns with the highest discrepancy between prehospital and clinical diagnoses being found in neurological disorders [4]. The acute management of difficult patient groups with non-specific symptoms and the need for increased detection of non-convulsive status epilepticus in acutely comatose patients have been recognized but lack feasible solutions [29–31].

The absence of a “gold standard” in prehospital management of neurological patients leads to high individual and regional variability, complicating the design of appropriate practical training [15]. Many researchers propose to have found the best option for prehospital triage, reflected in a plethora of prehospital stroke scales [24, 32, 33]. In our survey, the most widely known and used was the FAST score and its add-ons (BE-FAST or G-FAST). Other scores were known by only a minority of participants and used by even less. To simplify prehospital management, one potential improvement could be incorporating the score with the highest validation and practicality into documentation protocols, requiring identified items for triage to be checked even if the score itself is not known [6, 34].

Participants suggested adding a validated stroke scale and updating the current simple FAST test to include balance and ocular changes. Eye assessment was felt to be under-represented with the current items “pupil size” and “direct light response”. Additionally, the need for items such as amnesia and the Paediatric Glasgow Coma Scale was clearly stated. Our survey revealed a low correlation between the performed assessment and the final documentation. Updating documentation protocols could address this gap. However, without validation and definitive standards for neurological assessment schemes in a prehospital setting, optimizing protocols is challenging [35]. In addition, speech recognition for documentation could shorten the time spent on prehospital documentation, allowing more focus on patient care [36]. Practical training is essential for the implantation of theoretical knowledge and assessment scales. A significant increase in knowledge and communication skills has been reported with simulation-enhanced learning for acute stroke patients [37]. This approach is beneficial for more than just acute stroke cases. The availability of continuing education offers more knowledge, increasing the safety and comfort of EMS personnel [25].

Differentiating between an ischaemic stroke and an intracranial haemorrhage was identified by the majority as the biggest challenge. This challenge is reflected in a wide range of research focusing on portable, non-invasive technologies, including near-infrared spectroscopy, transcranial ultrasound, electroencephalography, microwave tomographic imaging, volumetric impedance spectroscopy, portable CT and cranial accelometry [38, 39]. While current commercial products do not yet reflect these technological advances, they have the potential to improve prehospital times and diagnostic accuracy, ultimately improving outcomes for patients with intracranial haemorrhage by enabling selection of the appropriate hospital for treatment [40, 41].

In addition to technological diagnostic tools, there is emerging research on point-of-care testing for prehospital distinction between ischaemic and haemorrhagic stroke [42, 43]. This differentiation is important for target hospital selection, emphasizing the need for neurological centres with appropriate treatment options for both stroke types.

Participants identified various challenges beyond stroke, including paediatric patients, trauma, vertigo, headache, isolated focal deficits, infectious diseases affecting the nervous system, intoxication, psychiatric emergencies, acute flare-ups of chronic diseases and unclear or unknown symptoms. There are existing tools for prehospital triage with a focus on stroke, trauma, general undifferentiated patients and children, but they are not without limitations and lack homogenous recommendations [29, 44]. Advances in telecommunication, and digital documentation could provide reliable neurological assessments [45–47]. Implementation new technologies and evidence-based algorithms is urgently needed to address current challenges [48].

Limitations.

This study relies on voluntary participation, which my introduce bias. Additionally, the lack of a call to complete the study may have resulted in a decrease in data quantity towards the end of the survey. Another limitation was the availability of only one language during the study, resulting in a regional bias. Further research should be conducted with broader availability of the survey to reduce regional bias.

## Conclusion

Participants in our survey suggested more standardized practical training, continuing education with uniform guidelines and algorithms for all EMS personnel, not just emergency physicians. Moreover, the need for peer collaboration, feedback, and case sharing was emphasized. Digitalisation, telemedicine, and updated documentation protocols, including paediatric adaptations to accommodate current guidelines, were considered important. To conclude, we would like to quote the responses of two of our participants: “How about using the age of technology and digitalization. There are probably a thousand ways for support and improve in the prehospital environment. But we are writing on paper and driving around with broken and old cars and systems.” And “The relevance can only be recognized when the fundamentals are understood”.

## Electronic supplementary material

Below is the link to the electronic supplementary material.

Supplementary Material 1

Supplementary Material 2

## Data Availability

The datasets used and/or analysed during the current study are available from the corresponding author on reasonable request.

## Declarations

### Patient and public involvement statement

The access link for the study was publicly distributed through snowballing, social media and through a QR code on a promotional poster.
