# Correction: Prehospital neurological emergencies– a survey on the state of prehospital neurological assessment by emergency medical professionals

**DOI:** 10.1186/s12873-024-01138-z

**Published:** 2024-11-18

**Authors:** Vesta Brauckmann, Dominica Hudasch, Pascal Gräff, Torben Riecke, Gökmen Aktas, Jorge Mayor, Christian Macke

**Affiliations:** 1 Department of Trauma Surgery, Hannover Medical School, Carl-Neuberg-Straße 1, D-30625 Hannover, Germany; 2 Department of Neurology, Hannover Medical School, Carl-Neuberg-Straße 1, 30625 Hannover, Germany

**BMC Emergency Medicine (2024) 24:164**

10.1186/s12873-024-01076-w

The original article has been updated to change co-author, Dominica Hudasch’s Family Name to ‘Hudasch’ from ‘Ratuszny’.

