# Supplementary material for: Prehospital neurological emergencies– a survey on the state of prehospital neurological assessment by emergency medical professionals
Source: BMC Emerg Med. 2024 Sep 11;24:164. doi: 10.1186/s12873-024-01076-w (PMC11389461; doi:10.1186/s12873-024-01076-w)
Supplement: Supplementary file 1 — Supplementary Material 1 [file 12873_2024_1076_MOESM1_ESM.pdf]

B001. Which role in the emergency medical services (EMS) do you have?
- Emergency physician
- Emergency Medical Technician
- Paramedic
- Other

B003. How long have you been working in the EMS?
- < 3 years
- 3-5 years
- 5-10 years
- >10 years

B004. Which emergency vehicle do you mostly staff?
- Rescue Helicopter
- Emergency Physician Vehicle
- Ambulance
- Mobile Intensive Care Unit
- Other

B002. What is your specialty outside of EMS? (For Emergency Physicians)
- Internal Medicine
- Anaesthesia
- Surgery
- Neurology
- Other

AN14. How often do you encounter neurological emergencies?
- 0 %
- 20 %
- 40 %
- 60 %
- 80 %
- 100 %

AN15. Do you feel a sense of unease when dispatched for a neurological emergency? If yes, why.
- Yes
- No

AN16. Which neurological emergency do you regard as biggest challenge?

AN01. Rate the quality of your general neurological examination? From 1 (very good) to 6 (insufficient)

AN02. How secure do you feel while performing a neurological assessment? From 1 (very secure) to 6 (very insecure)

AN08. Do you have a fixed scheme you can use for performing a neurological examination?
- Yes
- No
- Don’t know

AN07. Are you familiar with any standard operating procedures (SOPs) for prehospital neurological assassment?=
- Yes
- No
- Don’t know

AN03. How regularly do you evaluate the patients orientation (person, time, place and situation)? From 1 (very regularly) to 6 (very irregularly)

AN04. How often do you get the impression that with “difficult” patients no neurological assessment takes place? (E.g. Demented or intoxicated patients, children) From 1 (very often) to 6 (very rare)

AN09. Do you know how to perform a neurological examination on patients with impaired consciousness?
- Yes
- No

AN10. How secure do you feel in performing a pediatric neurological examination? From 1 (very secure) to 6 (very insecure)

AN11. Do you change your neurological assessment when examining children?
- Yes
- No

AN12. Are you familiar with the Paediatric Glasgow Coma Scale?
- Yes
- No

SN01. What is relevant for you in your GENERAL neurological assessment?
- Glasgow Coma Scale
- AVPU (Alert, Verbal Response, Pain, Unresponsive)
- FOUR Score (Full Outline of UnResponsiveness)
- Circulatory, Motor, Sensory Testing
- Pupillary Light Response
- Visual Activity
- Orientation
- FAST (Facial drooping, Arm weakness, Speech difficulties, Time to call emergency services)
- Speech
- Asymmetry
- Arm Drift Exam

- Facial Expressions
- Strength Grade
- Reflexes
- Pathological Reflexes
- Sensory Function
- Patient History
- Other

SN02. What do you pay attention to when assessing pupils?
- Size
- Direct light reflex
- Consensual light reflex
- Isocoria
- Shape
- Other

ST01. What is relevant for you in a specific stroke assessment?
- Orientation
- Speech
- Facial Symmetry
- Motoric Function of the Extremities
- Consciousness
- Obeying of Commands
- Coordination
- Occulomotoric
- Sensory Function
- Other

ST02. Are you familiar with specific tests / scales for stroke assessment? If yes, do you use them? (known / being used / not used / not known)
- FAST-Test (Facial Drooping, Arm weakness, Speech difficulties, Time to call emergency Services)
- RACE-Scale (Rapid Arterial Occlusion Evaluation)
- CPSS-Scale (Cincinnati Prehospital Stroke Scale)
- Other specific tests (e.g. NIHSS-Scale National Institute of Health Stroke Scale)

SE01. Have you ever seen a status epilepticus?
- Yes
- No
- I don’t know

SE02. Do you know what the difference is between a seizure and a status epilepticus?
- Yes
- No
- I don’t know

SE03. Do you know the difference between focal and generalized seizures?

- Yes
- No
- I don’t know

SE04. Do you need to treat every epileptic seizure?
- Yes
- No
- I don’t know

AN06. How useful do you find a prehospital neurological assessment? (From 1 (very useful) to 6 (very useless)

AN05. In your experience; How high is the correlation between the documentation and the actually performed examination? From 1 (very high) to 6 (very low).

AN13. How suitable do you think is the documentation for neurological emergencies? From 1 (very suitable) to 6 (Very unsuitable).

LF02. Would you want more training initiatives for a prehospital neurological assessment?
- Yes
- No
- Maybe
- I don’t know

LF03. Which kind of support would you need to help you improve prehospital neurological examination?
