# Supplementary figures and images for: Prehospital neurological emergencies– a survey on the state of prehospital neurological assessment by emergency medical professionals
Source: BMC Emerg Med. 2024 Sep 11;24:164. doi: 10.1186/s12873-024-01076-w (PMC11389461; doi:10.1186/s12873-024-01076-w)

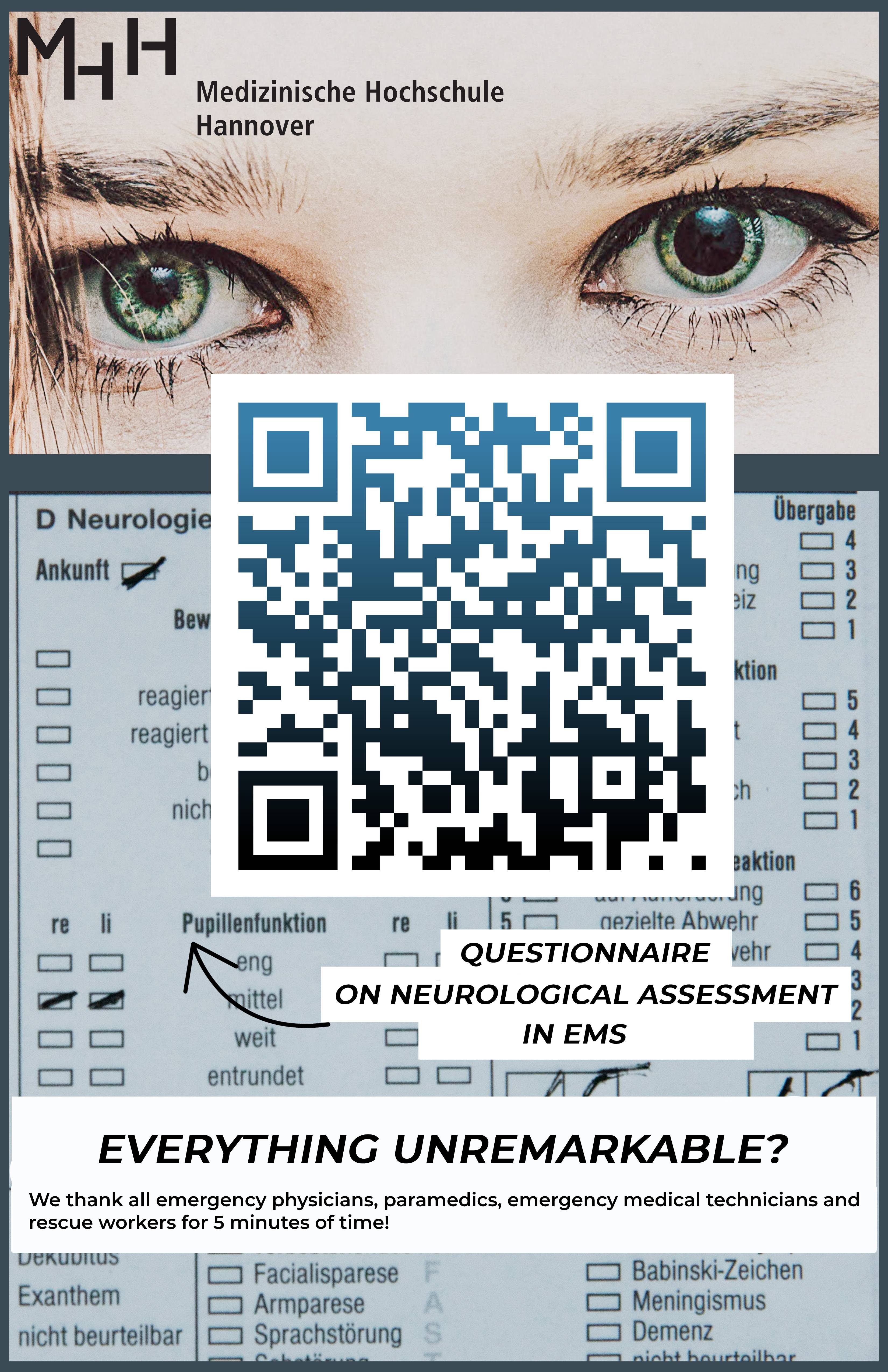

Supplement: Supplementary file 2 — Supplementary Material 2 [file 12873_2024_1076_MOESM2_ESM.jpg]
